# Maternal pregnancy-related anxiety and children’s physical growth: the Ma’anshan birth cohort study

**DOI:** 10.1186/s12884-023-05711-5

**Published:** 2023-05-25

**Authors:** Jixing Zhou, Shanshan Zhang, Yuzhu Teng, Jingru Lu, Yufan Guo, Shuangqin Yan, Fangbiao Tao, Kun Huang

**Affiliations:** 1 Department of Maternal, Child and Adolescent Health, School of Public Health, Anhui Medical University, Hefei, China; 2 Key Laboratory of Population Health Across Life Cycle (AHMU), MOE, Hefei, 230032 China; 3 NHC Key Laboratory of study on abnormal gametes and reproductive tract, Hefei, 230032 China; 4 Anhui Provincial Key Laboratory of Population Health and Aristogenics, Hefei, 230032 China; 5 Maternal and Child Health Care Center of Ma’anshan, No 24 Jiashan Road, Ma’anshan, 243011 Anhui China; 6 Scientific Research Center in Preventive Medicine, School of Public Health, Anhui Medical University, Hefei, 230032 Anhui China

**Keywords:** Pregnancy-related anxiety, Children, Body mass index, Body fat, Cohort

## Abstract

**Background:**

Epidemiological studies have identified maternal antenatal anxiety and several adverse birth outcomes, but limited studies have focused on the relationship with the long-term physical growth of children. The study aimed to assess the influence of maternal pregnancy-related anxiety on physical growth in children at different exposure periods during pregnancy.

**Methods:**

3,154 mother-child pairs were included based on the Ma’anshan birth cohort study. Maternal prenatal anxiety was obtained by administering a questionnaire using the pregnancy-related anxiety questionnaire (PRAQ) scale during the 1st, 2nd and 3rd trimesters of pregnancy. Body fat (BF) (48 to 72 months) and Body Mass Index (BMI) (birth to 72 months) were collected repeatedly for children. Group-based trajectory models were applied to fit the different trajectories of BMI and BF.

**Results:**

Maternal anxiety in the 2nd (OR = 0.81; 95% CI: 0.68 to 0.98; P < 0.025) and 3rd (OR = 0.80; 95% CI: 0.67 to 0.97; P = 0.020) trimesters was associated with a decreased risk of rapid weight gain (RWG) in the first year of life. Children aged 48 to 72 months of mothers with anxiety in the 3rd trimester had lower BMI (β = -0.161; 95% CI, -0.293 to -0.029; P = 0.017) and BF (β = -0.190; 95% CI, -0.334 to -0.046; P = 0.010), and these children were less likely to develop a very high BMI trajectory (OR = 0.54; 95% CI: 0.34 to 0.84; P = 0.006), and a high BF trajectory (OR = 0.72; 95% CI: 0.53 to 0.99; P = 0.043). Similar associations were found between maternal anxiety in both 2nd and 3rd trimesters and children’s physical growth.

**Conclusions:**

Offspring of mothers with prenatal anxiety in the 2nd and 3rd trimesters predicts poorer growth in infancy and preschool age. Early improvement and treatment of prenatal anxiety could benefit physical health and development in early childhood.

**Supplementary Information:**

The online version contains supplementary material available at 10.1186/s12884-023-05711-5.

## Introduction

Anxiety is a major health problem in clinical and public health areas. It predisposes women, particularly those who undergo severe physical and psychological changes during pregnancy, to anxiety and depressive symptoms [1]. A study including 34 countries indicated that the average prevalence of anxiety during pregnancy was 18.2%, 19.1%, and 24.6% in the 1st, 2nd, and 3rd trimesters, respectively [2].

Anxiety during pregnancy has a negative impact on both the mom’s and the child’s health. Prenatal anxiety is associated with a preference for cesarean delivery in pregnant women [3], a higher risk of eating disorders [4], a higher incidence of depression [5], deterioration in sleep quality [6] and an increased risk of suicide [7]. It may also raise the likelihood of negative temperament [8], emotional and behavioral difficulties [9], and allergy disorders [10, 11] in offspring.

Prenatal anxiety is increasingly being recognized as a possible risk factor for adverse birth outcomes such as preterm birth, low birth weight and preeclampsia [12, 13]. However, just a few cohort studies have looked at the links between maternal anxiety and offspring’s subsequent physical growth, and the results have been equivocal [14–18]. The Danish National Birth Cohort study discovered that maternal anxiety at 16 and 30 weeks of gestation was not connected with children’s overweight at age 7, but women who were concerned about delivery or child health had a slightly higher risk of becoming overweight [17]. The Canadian cohort All Our Families (AOF) observed no direct association between prenatal anxiety and child weight status at 24 months [18]. The Avon cohort (ALSPAC) research found a 0.2 kg/m^2^ rise in BMI from 25 to 31 months among children of anxious mothers at 18 and 32 weeks of gestation, as well as 2 and 21 months postpartum [15]. The Generation R findings revealed no relationship between mother anxiety symptoms at 20 weeks of pregnancy and child overweight/obesity at 4 years of age [14]. However, with this cohort’s 10-year follow-up, an increased risk of children’s overweight/obesity was observed [16]. In addition, no studies have focused on the association of maternal anxiety with rapid weight gain in early childhood.

Pregnancy-related anxiety, characterized by specific fears, worries and anxieties about pregnancy and its outcome [19], and its association with poor birth outcomes and obstetric and pediatric risk factors is higher than general anxiety and depression [20, 21]. Furthermore, given the peculiarities of early childhood physical development and the volatility of BMI in early childhood, the influence of psychological stress during pregnancy on children’s physical development may differ depending on age.

Limited knowledge is available on the relationship between maternal antenatal anxiety and children’s physical growth, especially in the Chinese population, where evidence is lacking. Thus, we hypothesized that maternal prenatal anxiety would have a long-term effect on offspring’s physical growth in childhood and that there may be a critical window for this effect. We aimed to assess the impact between maternal pregnancy-related anxiety and children’s physical growth, and identifying the key time window and possible cumulative effects.

## Materials and methods

### Study design

Data from the Ma’anshan birth cohort (MABC) study in China served as the basis for this research. Between May 2013 and September 2014, 3,474 pregnant women were recruited at their first prenatal visit at Maternal and Child Health Hospital in Ma’anshan. The research procedure has received approval from Anhui Medical University’s ethics and research committees (No. 20,131,195). Before enrolling in the study, the participants provided their written informed permission.

### Study participants

**Fig. 1:**
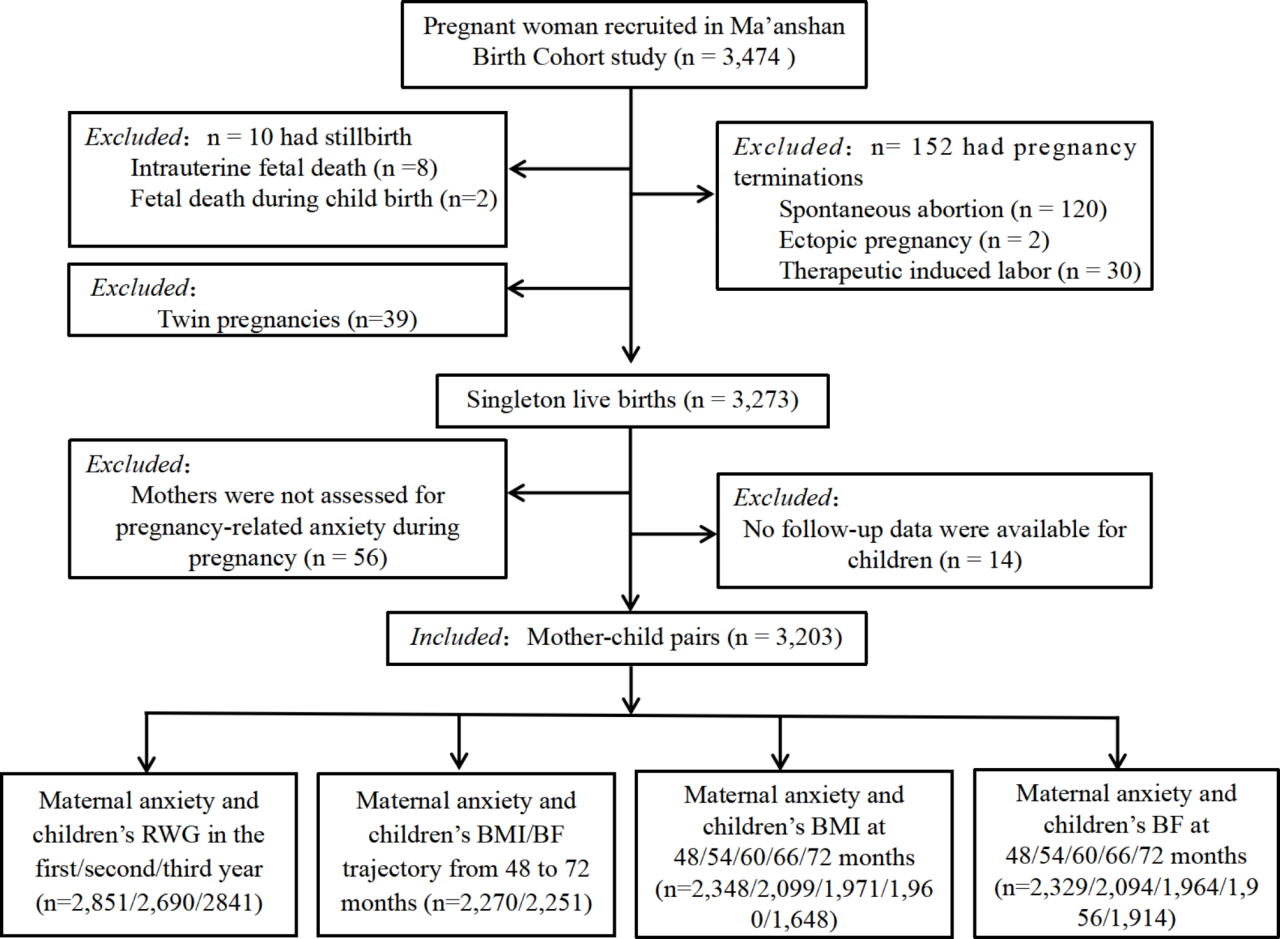
The follow-up diagram of the present study population

Inclusion criteria for pregnant women included: above the age of 18, within 14 gestational weeks; living in Ma’anshan city; willing to come to the center for antenatal checkups and delivery; without existing mental illness and able to understand and complete the questionnaire. We included a total of 3,273 singleton live births after excluding multiple pregnancies and adverse pregnancy outcomes, including embryonic arrest, stillbirth, spontaneous abortion, ectopic pregnancy and therapeutic abortion (n = 201). We further excluded women with no data for single pregnancy anxiety (n = 56) and children with no available physical data (n = 8). Finally, 3,154 mother-child pairs were included in the current study. The detailed flow chart of the mother-child pair selection is shown in Fig. 1.

### Pregnancy-related anxiety

Using the Pregnancy-Related Anxiety Questionnaire (PRAQ) [22], maternal anxiety in the 1st trimester (interquartile range: 8 to 11 weeks), 2nd trimester (interquartile range: 25 to 26 weeks), and 3rd trimester were evaluated (interquartile range 33 to 34 weeks). Thirteen items make up the screening scale, divided into three subscales: anxiety for the health of the woman herself (six items), anxiety for the health of the fetus (five items), and anxiety for childbirth (two items). For each question, women were asked to rate their level of anxiety on a scale of 1 to 4: “never worried”, “occasionally worried”, “often worried”, and “always worried”. The higher the score, the more severe the woman’s pregnancy-related anxiety symptoms. The total score of the scale ranged from 13 to 52. When the overall score was ≥ 24, women were classified as experiencing pregnancy-related anxiety. Cronbach’s coefficients were 0.81, 0.64, 0.78, and 0.74 for the total questionnaire and the three subscales, respectively [22]. The questionnaire was retested three weeks after the first-round test, and the retest reliability coefficients were 0.79, 0.67, 0.75, and 0.76, respectively [3]. Confirmatory factor analysis showed that the values of the goodness of fit index, normed fit index, relative fit index, comparative fit index and root mean square error of approximation were 0.949, 0.897, 0.871, 0.904, and 0.070, respectively [23]. The scale has been used to screen for and study pregnancy-related anxiety in many areas of China.

### Early childhood physical growth

The physical growth of the children was repeatedly evaluated from birth to 6 years of age. Five follow-up visits were made in the first year, i.e., at 42 days, 3 months, 6 months, 9 months, and 12 months, after delivery. Subsequently, the children underwent anthropometric measurements every six months until 6 years of age. Anthropometric measurements were performed by pediatric health professionals at Ma’anshan Maternal and Child Health Hospital.

The height and weight were measured at 16 follow-up visits from birth to 72 months. The body weight of the infants (birth to 12 months of age) was measured with a pan-type lever scale of 0.01 kg; and the weight of the toddlers (12 months to 30 months of age) was measured with a seated lever scale of 0.05 kg. The length of the children from birth to 30 months was evaluated with a standard measuring bed, accurate to 0.1 cm; and height and weight in children aged 3 to 6 years were measured with a mechanical height and weight scale (model: RRZ-50-RP), accurate to 0.1 kg for weight and 0.1 cm for height, respectively. Children were asked to remove their shoes and hats and wear light clothing during the measurements. The BMI was calculated using the following formula: BMI = weight (kg)/height (m^2^). Body fat (BF) was evaluated at 48, 54, 60, 66, and 72 months of age and was measured using an InBody J20 (Bioelectrical Impedance Fatness Analyzer). The test requires the child to cooperate and remain stationary for 1–2 min to obtain the body fat content.

The z-scores for BMI-for-age and weight-for-age (BMIZ; WAZ) children were identified and categorized (normal weight; overweight; overweight; underweight) using World Health Organization (WHO) Child Growth Standards and Growth Reference Data [24]. For the BF z-score, conversions were performed based on the included population.

### Early growth patterns in children

#### Rapid weight gain during infancy

The level of weight gain in children depended on the difference in WAZ between birth and 12/24/36 months (∆z-scores = z-scores 12/24/36 months - z-scores 0 months). We used the criteria for rapid weight gain (RWG) in infancy, defined as a change in weight SD score > + 0.67 from birth to 12/24/36 months, which is the most common and broadly accepted definition of RWG [25]. We classified the level of early weight gain into RWG (Δz-scores > 0.67) and no rapid weight gain (NRWG) (Δz-scores ≤ 0.67).

#### Child BMI and BF trajectory from 48 to 72 months of age

Group-based trajectory modeling (GBTM) was used to calculate BMI and BF trajectories for children aged 48 to 72 months. Given the current lack of reference standards for body composition in Chinese preschoolers, we considered trajectory fitting with raw values of physical indicators. Children with at least three BMI or BF values were permitted to examine quadratic trajectory models. The latent trajectory patterns of BMI rise in longitudinal BMI and BF data were identified using GBTM. Maximum likelihood techniques were implemented for parameter estimation and model fitting. Bayesian Information Criterion (BIC) and similarity trajectory shapes were used to identify the appropriate number of trajectory groups for different trajectories and to represent the appropriate functions for various trajectories in GBTM. Each person was assigned to the group with the most likely trajectory. Each person was assigned to the group with the most likely trajectory. In addition, we also fitted trajectories according to both BMI z-score ((based on WHO criteria) and BF z-score (based on the population included in this study).

### Covariates

Combining literature review and directed acyclic graphs (DAGs), we were able to find possible confounders (Figure S1). The confounders included maternal age, race (Han or others), family monthly income per capita (< 2500, 2500–4000, or > 4000 RMB), residence (urban or rural areas), parental education level (junior high school or below, senior middle school, junior college, or bachelor degree or above), maternal pre-pregnancy BMI, parity (0, ≥ 1), maternal metabolic dysfunctions (yes or no), alcohol use (yes or no), and tobacco use during pregnancy (yes or no), and father’s BMI. Children’s sex (boys or girls), birth weight, gestational age, breastfeeding duration, main caregivers before 3 years, average outdoor activity time, parenting style score and children’s diet were considered important covariates affecting offspring physical growth.

Women’s height and weight were measured at the first prenatal visit, and BMI was calculated according to the formula BMI (kg/m^2^) = weight (kg) / [height (m)]^2^ and considered as pre-pregnancy BMI. Maternal age, residence, race, parental education levels, family monthly income per capita, parity, and father’s BMI were collected by questionnaire during recruitment. Medical notes were used to extract data on maternal metabolic disorders, parity, children’s sex, birth weight and gestational age. Maternal metabolic dysfunctions included hypertensive disorders during pregnancy, gestational diabetes, and other metabolic dysfunctions (thyroid dysfunction, severe anemia, and polycystic ovary syndrome). Women with one or more of these disorders were defined as having maternal metabolic dysfunctions. Three-monthly questionnaires were used to gather information on alcohol and tobacco use during pregnancy.

In addition, data on breastfeeding duration (months), main caregivers before 3 years, average outdoor activity time, parenting style score and children’s diet were collected for sensitivity analyses.

### Statistical analysis

SPSS was used for all analyses (IBM, version 23.0). Statistical tests were conducted on a two-sided basis, with statistical significance defined as a P-value < 0.05. The normality of the distribution of the continuous variable was examined using the Shapiro-Wilk normality test. Participants’ demographic characteristics were reported as the mean ± standard deviation (*SD*) or percentage (*n %*). A bivariate analysis of baseline variables and covariates between the groups with and without maternal anxiety was performed.

Several analyses were conducted to determine the relationship between maternal anxiety and offspring’s physical development. Considering the impact of prenatal anxiety on offspring’s physical development and the possible critical period or the cumulative effect of long-term effects, we conducted the following categorical exposure analysis. First, we classified four categories according to the presence or absence of pregnancy-related anxiety: 1st trimester (yes vs. no), 2nd trimester (yes vs. no), 3rd trimester (yes vs. no), and all three trimesters (yes vs. no). Second, we explored the association of new-onset anxiety in the 2nd trimester (vs. no anxiety in the first two trimesters) and new-onset anxiety in the 3rd trimester (vs. no anxiety in all three trimesters) with offspring’s physical development. Third, taking into account the results of the previous analysis, we performed a post hoc analysis—of the association between simultaneous 2nd- and 3rd-trimester anxiety (vs. no anxiety both in the 2nd- and 3rd trimesters) and offspring’s physical growth.

Logistic regression models were used to explore the association between maternal antenatal anxiety and children’s RGW, BMI/BF trajectory and weight status. For the BMI and BF trajectory, the normal BMI/BF trajectory was used as the reference group. For weight status, the normal weight was used as the reference group.

Longitudinal analyses were performed using generalized estimating equations (GEEs) with random intercepts for each subject to account for the correlation between repeated observations within subjects between exposure to pregnancy-related anxiety and offspring’s physical growth (BMI and BF) from 48 months to 72 months of age. Furthermore, considering the characteristics of children’s early physical development, we used multiple linear regression models to compare the anthropometric outcomes (BMI and BF) of offspring exposed and not exposed to pregnancy-related anxiety at each follow-up time point. And the results are reported as unstandardized *B* coefficients with *95% CIs* after controlling for covariates.

Finally, to test the reliability of our findings, we performed three sensitivity analyses. First, maternal anxiety during pregnancy and children’s subsequent anthropometric growth may be mediated by sex, gestational age, and birth weight. Therefore, we further adjusted the birth weight and gestational age in the sensitivity analysis. Second, breastfeeding duration, main caregivers before 3 years, average outdoor activity time, parenting style, children’s diet are important factors for children’s physical growth. To improve the accuracy of the results, these were taken into consideration as the precision variable. Third, we explored the association of maternal pregnancy-related anxiety with children’s BMI z-score and BF z-score trajectories at 48 to 72 months of age.

## Results

### Characteristics of the study population

The basic characteristics of the included population (n = 3,203) are presented in Table 1. The pregnant women had an average age of 26.4 years, and their pre-pregnancy BMI was 20.9 kg/m^2^. Most mothers (90.1%) were nulliparous and Han Chinese (98.4%). The included pregnant women mainly lived in the urban area (60.9%). During pregnancy, the rates of maternal smoking and alcohol consumption were 0.2% and 8.1%, respectively. The mean birth weight of the children was 3363.2 g, and their gestational age was 39.0 weeks. The prevalence of anxiety in the 1st trimester, 2nd trimester, and 3rd trimester was 20.8% (487/2344), 23.0% (708/3080), and 22.9% (695/3031), respectively.

**Table 1:** Characteristics of the included participants in the study [Mean ± SD or n (%)]

Characteristics	Total participants (N = 3,203)
	Mean (SD) or N (%)
***Maternal characteristics***	
Age, years	26.4(3.6)
Race	
Han	3151(98.4)
Others	52(1.6)
Residence	
Rural	1252(39.1)
Urban	1951(60.9)
Parity	
Multipara	317(9.9)
Nulliparous	2886(90.1)
Education level	
Junior high school or below	637(19.9)
Senior middle school	717(22.4)
Junior college	998(31.2)
Bachelor degree or above	851(26.6)
Family monthly incomes (RMB^)^	
< 2500	838(26.2)
2500–4000	1375(42.9)
> 4000	990(30.9)
Alcohol use	
No	2942(91.9)
Yes	261(8.1)
Tobacco use	
No	3198(99.8)
Yes	5(0.2)
Pre-pregnancy BMI, kg/m^2^	20.9(2.9)
Pregnancy complications	
No	2649(82.7)
Yes	554(17.3)
*Father’s characteristics*	
BMI, kg/m^2^	23.3(3.6)
Education level	
Junior high school or below	470(14.7)
Senior middle school	886(27.7)
Junior college	870(27.2)
Bachelor degree or above	977(30.5)
***Child characteristics***	
Sex	
Female	1624(50.7)
Male	1579(49.3)
Gestational age, wk	39.0(1.3)
Birth weight, g	3363.2(446.6)

There were statistically significant differences in the proportion of mothers with pregnancy-related anxiety during pregnancy in terms of place of residence, parental education level, alcohol consumption and parity compared to mothers without anxiety during pregnancy (Table S1).

### Information on physical growth outcomes

**Fig. 2:**
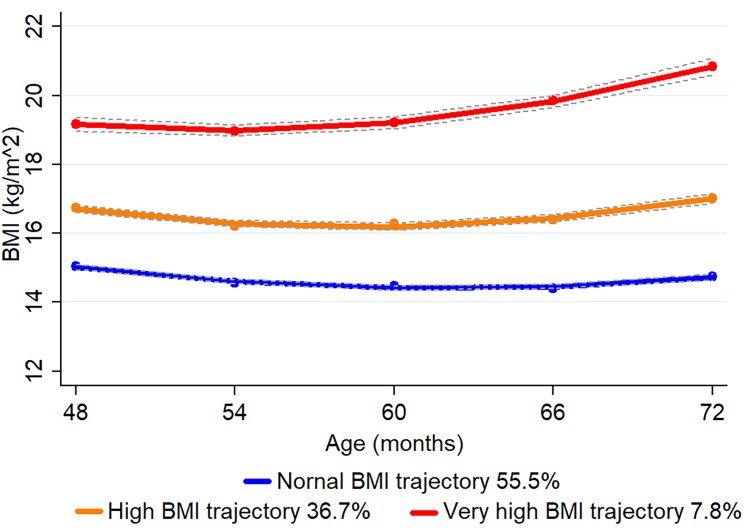
Body mass index (BMI) trajectories of children from 48 to 72 months

**Fig. 3:**
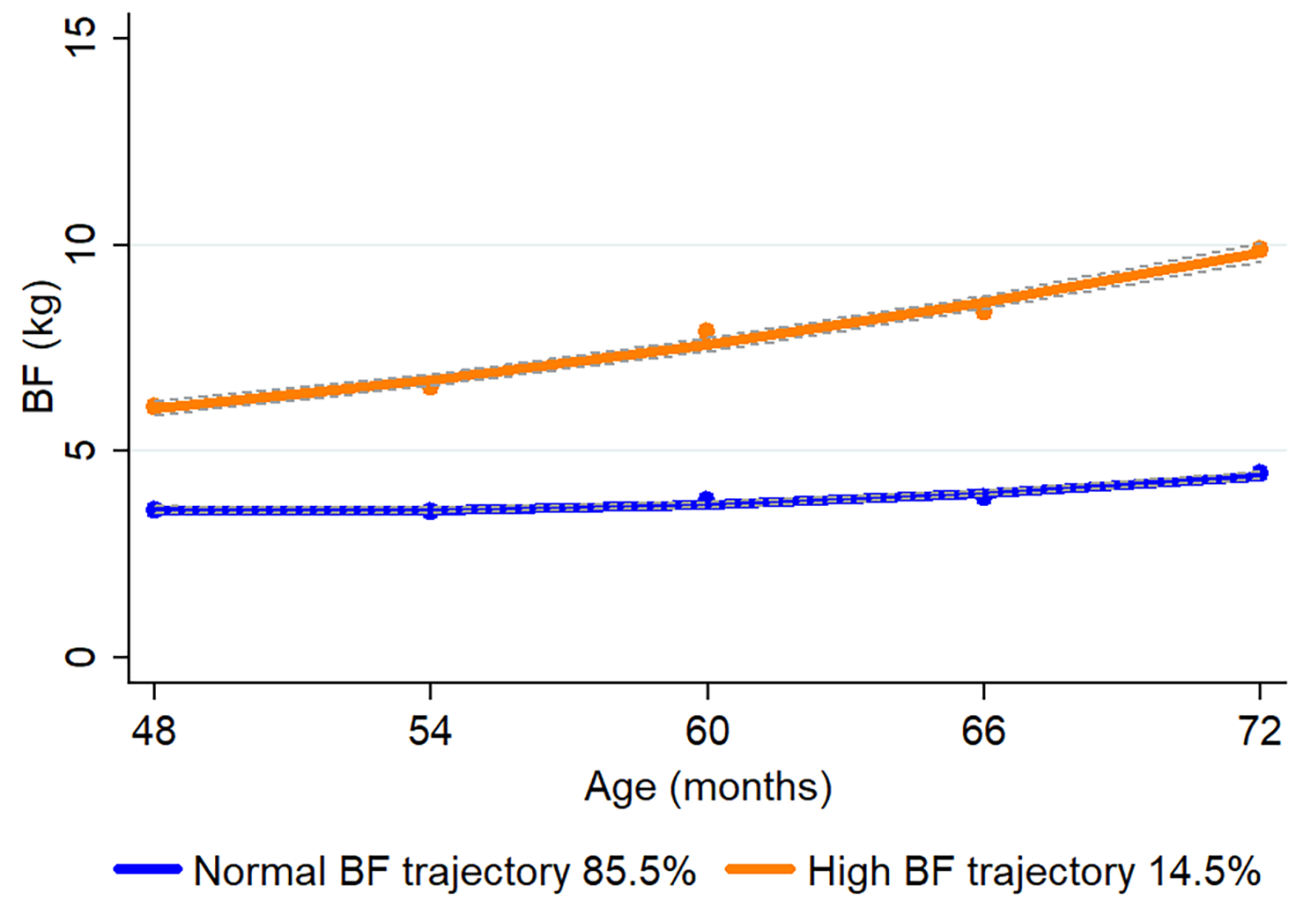
Body fat (BF) trajectories of children from 48 to 72 months

The prevalence of RGW in children was 45.6% (n = 1,300), 38.3% (n = 1,030) and 37.5% (n = 1,066) in the first year, the first two years, and the first three years, respectively. For childhood BMI trajectories from 48 to 72 months, we fitted three BMI trajectories: normal BMI trajectory (55.5%, n = 1260), high BMI trajectory (36.7%, *n* = 833), and very high BMI trajectory (7.8%, *n* = 177) (Fig. 2). For childhood BF trajectories from 48 to 72 months, we fitted two trajectories: normal BF trajectory (85.5%, *n* = 1925) and high BF trajectory (14.5%, *n* = 326) (Fig. 3).

For childhood BMI z-score trajectories from 48 to 72 months, we fitted three trajectories: normal BMI trajectory (50.7%), high BMI trajectory (13.1%), and low BMI trajectory (36.2%) (Figure S2). For childhood BF z-score trajectories from 48 to 72 months, we fitted two trajectories: normal BF trajectory (85.5%) and high BF trajectory (14.5%) (Figure S2).

### Maternal pregnancy-related anxiety and early RWG

Table 2 shows the relationships between maternal pregnancy-related anxiety and children’s RWG.

**Table 2:** Associations between maternal pregnancy-related anxiety and children’s RWG.

Time of maternal prenatal anxiety	RWG(0–12 months)			RWG(0–24 months)			RWG(0–36 months)	
	*OR (95% CI)*	*P-value*		*OR (95% CI)*	*P-value*		*OR (95% CI)*	*P-value*
***Model 1***								
First trimester	0.97(0.78,1.21)	0.803		1.01(0.80,1.27)	0.937		1.02(0.81,1.27)	0.891
Second trimester	**0.81(0.68,0.98)**	0.025		0.89(0.73,1.08)	0.224		0.94(0.78,1.13)	0.506
Third trimester	**0.80(0.67,0.97)**	0.020		0.91(0.75,1.11)	0.354		0.95(0.79,1.15)	0.588
At least one trimester during pregnancy	0.89(0.75,1.05)	0.168		0.97(0.81,1.16)	0.719		1.00(0.84,1.19)	0.968
New-onset anxiety in the Second trimester	**0.68(0.50,0.92)**	0.011		**0.69(0.50,0.96)**	0.025		0.84(0.62,1.14)	0.259
New-onset anxiety in the Third trimester	0.85(061,1.20)	0.365		1.12(0.78,1.60)	0.548		1.09(0.76,1.55)	0.655
Both Second and Third trimesters	**0.72(0.58,0.90)**	0.004		0.81(0.64,1.02)	0.074		0.91(0.72,1.14)	0.387
***Model 2***								
First trimester	0.98(0.76,1.25)	0.849		1.03(0.79,1.34)	0.844		1.02(0.79,1.32)	0.858
Second trimester	**0.81(0.66,1.00)**	0.047		0.92(0.73,1.15)	0.463		0.95(0.77,1.18)	0.644
Third trimester	**0.81(0.65,0.99)**	0.048		0.96(0.77,1.21)	0.743		1.01(0.81,1.26)	0.936
At least one trimester during pregnancy	0.85(0.70,1.03)	0.106		0.96(0.77,1.18)	0.668		1.00(0.81,1.22)	0.975
New-onset anxiety in the Second trimester	**0.60(0.43,0.84)**	0.003		**0.62(0.42,0.90)**	0.013		0.78(0.55,1.12)	0.181
New-onset anxiety in the Third trimester	0.90(0.61,1.33)	0.595		1.29(0.85,1.96)	0.239		1.30(0.86,1.95)	0.218
Both Second and Third trimesters	**0.71(0.55,0.91)**	0.007		0.82(0.62,1.09)	0.172		0.92(0.71,1.20)	0.533
***Model 3***								
First trimester	0.99(0.77,1.26)	0.934		1.01(0.77,1.32)	0.951		1.00(0.78,1.30)	0.980
Second trimester	**0.81(0.66,1.00)**	0.048		0.91(0.72,1.14)	0.395		0.94(0.75,1.16)	0.556
Third trimester	**0.81(0.65,1.00)**	0.049		0.94(0.75,1.19)	0.610		1.00(0.80,1.25)	0.993
At least one trimester during pregnancy	0.87(0.71,1.05)	0.150		0.93(0.75,1.15)	0.487		0.97(0.79,1.19)	0.794
New-onset anxiety in the Second trimester	**0.61(0.43,0.85)**	0.004		**0.58(0.39,0.85)**	0.005		0.76(0.53,1.09)	0.131
New-onset anxiety in the Third trimester	0.94(0.64,1.39)	0.768		1.23(0.80,1.88)	0.350		1.27(0.84,1.93)	0.255
Both Second and Third trimesters	**0.71(0.55,0.91)**	0.007		0.80(0.60,1.06)	0.117		0.91(0.70,1.19)	0.482

Model 1: adjusted for maternal age, residence, race, family monthly income per capita, maternal education level, father’s education level, maternal pre-pregnancy BMI, father’s BMI, parity, maternal metabolic dysfunctions, alcohol use, and tobacco use during pregnancy

Model 2: adjusted for covariates in model 1 + birth weight, gestational age, children’s sex

Model 3: adjusted for covariates in model 2 + breastfeeding duration, main caregivers before 3 years, average outdoor activity time, parenting style score, and children’s diet

After adjusted for confounders, we found that infants born to mothers with anxiety in the 2nd (*OR* = 0.81; *95% CI*: 0.68 to 0.98) and 3rd (*OR* = 0.80; *95% CI*: 0.67 to 0.97) trimesters had a decreased risk of RWG from birth to 12 months. Maternal new-onset anxiety in the 2nd trimester was associated with a decreased risk of RWG from birth to 12 months (*OR* = 0.68; *95% CI*: 0.50 to 0.92) and from birth to 24 months (*OR* = 0.69; *95% CI*: 0.50 to 0.96). Furthermore, we also found that infants born to mothers with anxiety both in the 2nd and 3rd trimesters had a decreased risk of RWG from birth to 12 months (*OR* = 0.72; *95% CI*: 0.58 to 0.90).

### Maternal pregnancy-related anxiety and early childhood physical growth by GEEs

Table 3 shows the relationships between maternal anxiety during pregnancy and early childhood anthropometric outcomes (BMI and BF).

**Table 3:** Associations between maternal pregnancy-related anxiety and children’s BMI and BF levels from 48 to 72 months of age

Time of maternal prenatal anxiety	Age of children	BMI			BF	
		*β(95%CI)*	*P-value*		*β(95%CI)*	*P-value*
*Model 1*						
First trimester	48–72 months	0.035(-0.139,0.210)	0.691		0.029(-0.165,0.222)	0.770
Second trimester		-0.063(-0.202,0.076)	0.376		-0.091(-0.243,0.061)	0.240
Third trimester		**-0.161(-0.293,-0.029)**	0.017		**-0.190(-0.334,-0.046)**	0.010
At least one trimester during pregnancy		-0.013(-0.145,0.118)	0.844		-0.033(-0.181,0.114)	0.659
New-onset anxiety in the Second trimester		-0.157(-0.366,0.052)	0.140		-0.202(-0.427,0.023)	0.079
New-onset anxiety in the Third trimester		-0.148(-0.394,0.099)	0.241		-0.168(-0.436,0.100)	0.218
Both Second and Third trimesters		**-0.181(-0.334,-0.028)**	0.020		**-0.219(-0.384,-0.054)**	0.009
*Model 2*						
First trimester	48–72 months	0.010(-01.64,0.184)	0.910		0.029(-0.165,0.222)	0.770
Second trimester		-0.067(-0.205,0.070)	0.336		-0.091(-0.243,0.061)	0.240
Third trimester		**-0.156(-0.285,-0.027)**	0.018		**-0.192(-0.336,-0.044)**	0.010
At least one trimester during pregnancy		-0.026(-0.156,0.103)	0.693		-0.033(-0.181,0.114)	0.659
New-onset anxiety in the Second trimester		-0.138(-0.335,0.059)	0.170		-0.202(-0.427,0.023)	0.079
New-onset anxiety in the Third trimester		-0.157(-0.397,0.083)	0.200		-0.168(-0.436,0.100)	0.218
Both Second and Third trimesters		**-0.178(-0.328,-0.028)**	0.020		**-0.219(-0.384,-0.054)**	0.009
*Model 3*						
First trimester	48–72 months	0.014(-0.161,0.190)	0.871		0.029(-0.165,0.085)	0.770
Second trimester		-0.066(-0.205,0.072)	0.348		-0.091(-0.243,0.061)	0.240
Third trimester		**-0.153(-0.284,-0.023)**	0.021		**-0.190(-0.334,-0.046)**	0.010
At least one trimester during pregnancy		-0.018(-0.151,0.114)	0.785		-0.033(-0.181,0.114)	0.659
New-onset anxiety in the Second trimester		-0.143(-0.342,0.056)	0.160		-0.202(-0.427,0.023)	0.079
New-onset anxiety in the Third trimester		-0.140(-0.381,0.101)	0.256		-0.168(-0.436,0.100)	0.218
Both Second and Third trimesters		**-0.183(-0.334,-0.033)**	0.017		**-0.219(-0.384,-0.054)**	0.009

Model 1: adjusted for maternal age, residence, race, family monthly income per capita, maternal education level, father’s education level, maternal pre-pregnancy BMI, father’s BMI, parity, maternal metabolic dysfunctions, alcohol use, and tobacco use during pregnancy

Model 2: adjusted for covariates in model 1 + birth weight, gestational age, children’s sex

Model 3: adjusted for covariates in model 2 + breastfeeding duration, main caregivers before 3 years, average outdoor activity time, parenting style score, and children’s diet

After adjusted for confounders, it was found to be that maternal anxiety in the 3rd trimester was correlated to lower BMI (*β* = -0.161; 95% CI: -0.293 to -0.029) and BF (*β* = -0.190; *95% CI*: -0.334 to -0.046) from 48 to 72 months.

Among the cumulative effects analyzed, we found that the offspring of mothers with anxiety both in the 2nd and 3rd trimesters had lower BMI (*β* = -0.181; *95% CI*: -0.334 to -0.028) and BF (*β* = -0.219; *95% CI*: -0.384 to -0.054).

### Maternal pregnancy-related anxiety and early childhood BMI trajectories

Table S2 presents the association between maternal antenatal anxiety and childhood BMI trajectories from 48 to 72 months.

Using the normal BMI trajectory as a reference, children born to mothers with anxiety in the 3rd trimester were related to a lower risk of very high BMI trajectory (*OR* = 0.54; *95% CI*: 0.34 to 0.84). Maternal anxiety with new-onset anxiety in the 3rd trimester was related to a lower risk of high BMI trajectory (*OR* = 0.63; *95% CI*: 0.41 to 0.97).

Furthermore, maternal anxiety both in the 2nd and 3rd trimesters was associated with a lower risk of very high BMI trajectory (*OR* = 0.53; *95% CI*: 0.30 to 0.92).

### Maternal pregnancy-related anxiety and early childhood BF trajectories

Table S3 displays the association between maternal antenatal anxiety and childhood BF trajectories from 48 to 72 months.

Using the normal BF trajectory as a reference, children of mothers who experienced prenatal anxiety in the 3rd trimester had a decreased risk of a high BF trajectory (*OR* = 0.72; *95% CI*: 0.53 to 0.99). Maternal anxiety both in the 2nd and 3rd trimesters had a decreased risk of high BF trajectory (*OR* = 0.65; *95% CI*: 0.44 to 0.95).

### Maternal pregnancy-related anxiety and early childhood physical growth in each time

For BMI, children of mothers who experienced anxiety during the third 3rd had lower BMI at 48 months (*β* = -0.201; *95% CI*: -0.373 to -0.029) and 60 months (*β* = -0.217; *95% CI*: -0.393 to -0.042). Furthermore, children of mothers with anxiety both in the 2nd and 3rd trimesters had lower BMI in age at 48 months (*β* = -0.244; *95% CI*: -0.451 to -0.038) Table S4).

For BF, it was found that children born to mothers with anxiety in the 3rd trimester had lower BMI aged 48 months (*β* = -0.169; *95% CI*: -0.330 to -0.009), 54 months (*β* = -0.232; *95% CI*: -0.410 to -0.054) and 60 months (*β* = -0.278; *95% CI*: -0.506 to -0.050). Maternal new-onset anxiety in the 2nd trimester had a lower BMI at 54 months (*β* = -0.291; *95% CI*: -0.558 to -0.025). Furthermore, children of mothers with anxiety both in the 2nd and 3rd trimesters had lower BMI at 48 months (*β* = -0.203; *95% CI*: -0.397 to -0.009) and 54 months (*β* = -0.231; *95% CI*: -0.444 to -0.018) of age (Table S5).

### Maternal pregnancy-related anxiety and children’s weight in each time

Table S6 presents the association between maternal antenatal anxiety and children’s weight status from 48 to 72 months. Children born to mothers with anxiety in the 3rd trimester of pregnancy had a lower risk of overweight and/or obesity at 48 to 66 months of age. Furthermore, children of mothers with anxiety both in the 2nd and 3rd trimesters also had a lower risk of overweight and/or obesity at 48 to 66 months of age.

### Sensitivity analysis

Sensitivity analyses 1–2 showed that our main results remained stable (Tables 2 and 3, Table S2-S6).

We also observed that children of maternal anxiety in the 3rd trimester and maternal anxiety both in the 2nd and 3rd trimesters had a lower risk of high BMI z-score trajectory and high BF z-score trajectory from 48 to 72 months (Table S7-S8).

## Discussion

The results of this birth cohort study support that maternal prenatal anxiety predicts lower levels of physical growth in children. Maternal anxiety in the 2nd and 3rd trimesters of pregnancy was associated with a lower risk of RWG in the first year of life. Furthermore, we observed that children born to mothers with anxiety in the 3rd trimester had lower BMI and BF from 48 to 72 months. When maternal anxiety in both 2nd and 3rd trimesters was observed, it was similarly associated with offspring’s physical growth.

In the 1st, 2nd, and 3rd trimesters of pregnancy, the overall prevalence of pregnancy-related anxiety was 20.8%, 23.0%, and 22.9%, respectively. This is roughly similar to the prevalence of pregnancy-related anxiety reported in other regions [26]. Our study revealed that the offspring of mothers with anxiety in the 2nd and 3rd trimesters are less prone to RWG in the first year of life. Similarly, a study from Pakistan found that infants of prenatally depressed mothers exhibited significantly more growth retardation than controls at both 6 and 12 months [27]. Another study of Latinx pregnant women also found that offspring of mothers with depression prenatally and at 4–6 weeks postpartum had less weight gain 2 years after birth [28].

The fetal origin hypothesis suggests that fetuses that experience malnutrition in utero usually develop intrauterine growth restriction while acquiring a thrifty phenotype with good energy efficiency through altered endocrine and metabolic mechanisms [29]. Typically, physical growth in school-age children is a continuation of growth patterns from birth outcomes and infancy. Evidence from epidemiological studies suggests that maternal prenatal anxiety is positively associated with prenatal growth retardation [12, 13]. Both cohorts conducted by our team at the Ma’anshan Maternal and Child Health Hospital suggest that maternal prenatal anxiety is associated with an increased risk of PTB and LBW/SGA [30, 31]. For example, the general response of the fetus to maternal nutritional deficiency is reduced wild growth, and if fetal malnutrition persists, the impaired growth trajectory may become irreversible. Such changes are clearly beneficial to the fetus and help optimize the use of limited [32].

Limited birth cohort studies have explored the association between maternal anxiety and long-term physical growth in offspring [14–18]. It is worth noting that the Generation R study found maternal anxiety symptoms at 20 gestational weeks were not associated with offspring’s overweight/obesity at 4 years of age [14] but associated with an elevated risk of overweight/obesity at age 10 [16]. According to an Australian study, high scores for stressful life events during pregnancy are correlated with BMI, overweight, and obesity in offspring at the age of 20 [33]. Our findings found that offspring with maternal antenatal anxiety in 2nd trimester and both 2nd and 3rd trimesters had lower mean BMI and BF between 4 and 6 years of age, but further analyses by time point revealed a more pronounced association at 48, 54, and 60 months, with this association almost disappearing at 66 and 72 months of age. Children’s age may be one of the important factors explaining this difference [14, 16]. It is well known that children’s growth patterns from birth to adulthood are constantly changing, especially around the onset of adiposity rebound (AR) [34] and puberty [35]. Ramel et al. also found that preterm infants had relatively lower fat-free mass and lower weight compared to full-term infants, but these differences gradually decreased with the change in age [36]. Furthermore, the present study found that the association of maternal prenatal anxiety with RGW and BMI/BF occurred in 2nd and/or 3rd trimesters of pregnancy, suggesting that maternal stress during pregnancy may have a window of effect on the physical growth of the offspring [37].

The “adipose tissue expandability” hypothesis proposed by Virtue and Vidal-Puig [38] assumes that the maximum capacity of adipose tissue to increase mass is limited and that this is determined on an individual basis by environmental and genetic factors [39]. In this hypothesis, insulin sensitivity will remain high as long as adipose tissue is able to expand. Once the point of maximal expansion of adipose tissue is reached, metabolic complications occur rapidly. Not only adipocyte size but also adipocyte number is important for adipose tissue accumulation [40]. Infants with a frugal phenotype tend to have more energy; however, these children tend to be leaner and have fewer adipocytes, which can lead to an energy overload of individual adipocytes. There is a limit to the maximum hypertrophy of individual adipocytes; therefore, this would be associated with the impaired expansiveness of the entire adipose tissue [41]. Biological differences in body composition across ethnic groups suggest that BMI and BF growth patterns may likewise differ in children early in life [42]. Furthermore, we noticed that most of the available studies on the positive association between anxiety/stress during pregnancy and offspring overweight/obesity are from developed countries and are mainly severely stressful events [43]. It is important to know that there are multiple differences in economic, cultural, early childhood life and dietary patterns in China compared to Western countries, which may influence maternal and infant care and early childhood physical development patterns.

Maternal anxiety or other persistent stress exposure and response during pregnancy may influence the offspring’s birth outcomes and later physical development through multiple complex physiological and behavioral pathways [44]. The function of the hypothalamic-pituitary-adrenocortical (HPA) axis is one of the most likely underlying processes which will trigger the release of stress hormones such as cortisol when individuals are suffering from stressful events [45, 46]. Anxiety during pregnancy is chronic stress that can reduce the activity of 11-beta-hydroxysteroid dehydrogenase type 2 (11β-HSD-2) in the placenta and increase the input of cortisol from the mother to the fetus [47, 48]. An appropriate increase in cortisol concentration is essential for the development and maturation of fetal organs [49]. However, chronic exposure to elevated glucocorticoids (GCs) may contribute to increased vascular resistance observed in fetal placental circulation, which is a hallmark of fetal growth restriction [50].

Our study has several advantages. To begin, this study is based on a prospective birth cohort. All data about exposure, outcome, and potential covariates were gathered prospectively. Secondly, maternal anxiety was assessed separately in the 1st, 2nd, and 3rd trimesters, which provides sufficient power to explore possible critical periods and dose-response relationships for the impacts of maternal anxiety during pregnancy on offspring’s physical development. Thirdly, the relationship between maternal anxiety and offspring’s anthropometric development is explored through multiple repeated measures, multiple methods and multiple indicators, which improves the reliability of the results. Lastly, we also adjusted several important covariates such as father’s BMI, father’s educational level, children’s birth weight and gestational week and postpartum breastfeeding duration, which improved the stability of the results.

However, the present study also has some limitations. First, the current cohort was only followed up to 6 years of age. Although we observed significant differences in BMI and BF levels and the risk of overweight/obesity, very high BMI trajectory, and high BF trajectory between anxious and non-anxious mothers in the third trimester from 48 to 72 of age, we do not know the trends after 72 months. But the MABC study is still being followed up, and future studies will continue to update the direction and intensity of this association. Second, we only used the mother’s self-reported pregnancy-related anxiety scale, and did not use other scales or clinical diagnosis to test the stability of the results. Because the use of self-reported anxiety scale may have introduced measurement inaccuracy. Third, we did not evaluate and control for maternal postnatal mood since the MABC research focused on the child’s growth rather than the mother. However, the brief toddler parenting style questionnaire contains entries on parent-child interaction as well as mental health, which largely reflects the level of mental health of mothers in the postpartum period. We have controlled for parenting behaviors in sensitivity analyses, and the results were consistent with the main analysis. Fourth, we did not collect several factors that may affect physical development, such as necrotizing enterocolitis, neonatal sepsis, asthma, infectious diseases, chorioamnionitis, and cardiovascular diseases, which limits the further exploration of the findings. However, we made five repeated measurements in childhood, which largely avoided the measurement error caused by a single measurement and reduced the interference of these diseases on the correlation between exposure and outcome. Moreover, we found that children with maternal anxiety in the 3rd trimester were less likely to develop a high BMI trajectory and a high BF trajectory at age 4–6 years. Although this association was observed, whether this effect was positive or negative remains to be argued.

## Conclusions

In conclusion, children born to maternal antenatal anxiety in 3rd trimester had a decreased risk of RWG in the first years of life and had a lower weight status (BMI and BF) in the preschool stage. Similar associations were found in the offspring of mothers with concurrent anxiety in 2nd and 3rd trimesters of pregnancy. Early improvement and treatment of prenatal anxiety may benefit early childhood physical health and development.

## Electronic supplementary material

Below is the link to the electronic supplementary material.

**Additional file 1:** Supplementary Figures and Tables

## Data Availability

The datasets used and/or analyzed in the current study are available upon reasonable request to the corresponding author.

## Abbreviations

MABC: The Ma’anshan birth cohort
DAG: Directed acyclic graph
BMI: Body mass index
BF: Body fat
SD: Standard deviation
OR: Odds ratio
MD: Mean difference
RWG: Rapid weight gain
GEEs: generalized estimating equations
