# Supplementary material for: Maternal pregnancy-related anxiety and children’s physical growth: the Ma’anshan birth cohort study
Source: BMC Pregnancy Childbirth. 2023 May 25;23:384. doi: 10.1186/s12884-023-05711-5 (PMC10210302; doi:10.1186/s12884-023-05711-5)
Supplement: Supplementary file 1 — Additional file 1: Supplementary Figures and Tables [file 12884_2023_5711_MOESM1_ESM.docx]

**Table S1** Comparison of the the basic characteristics between maternal pregnancy-related anxiety and maternal non-pregnancy-related anxiety [Mean ± SD or n (%)]

| **Characteristics** | **The groups with maternal anxiety during pregnancy (n=1102)** | **The groups without maternal anxiety during pregnancy(n=1472)** | **P-value** |
| --- | --- | --- | --- |
| *Maternal characteristics* |  |  |  |
| Age, years [Mean (SD)] | 25.9(3.4) | 26.6(3.7) | 0.561 |
| Race [n (%)] |  |  | 0.877 |
| Han | 1083(98.3) | 1448(98.4) |  |
| Others | 19(1.7) | 24(1.6) |  |
| Residence [n (%)] |  |  | 0.001 |
| Rural | 477(43.3) | 545(37.0) |  |
| Urban | 625(56.7) | 927(63.0) |  |
| Parity [n(%)] |  |  | <0.001 |
| Multipara | 81(7.4) | 171(11.6) |  |
| Nulliparous | 1021(92.6) | 1301(88.4) |  |
| Education level [n (%)] |  |  | <0.001 |
| Junior high school or below | 233(21.1) | 270(18.3) |  |
| Senior middle school | 284(25.8) | 303(20.6) |  |
| Junior college | 337(30.6) | 469(31.9) |  |
| Bachelor degree or above | 248(22.5) | 430(29.2) |  |
| Family monthly incomes (RMB^)^ [n (%)] |  |  | 0.074 |
| <2500 | 303(27.5) | 348(23.6) |  |
| 2500-4000 | 470(42.6) | 648(44.0) |  |
| >4000 | 329(29.9) | 476(32.3) |  |
| Alcohol use [n (%)] |  |  | <0.001 |
| No | 979(88.8) | 1377(93.5) |  |
| Yes | 123(11.2) | 95(6.5) |  |
| Tobacco use [n (%)] |  |  | 0.580 |
| No | 1100(99.8) | 1471(99.9) |  |
| Yes | 2(0.2) | 1(0.1) |  |
| Pre-pregnancy BMI, kg/m^2^ [Mean (SD)] | 20.8(2.8) | 20.9(2.8) | 0.805 |
| Pregnancy complications [n (%)] |  |  | 0.175 |
| No | 913(82.8) | 2162(84.0) |  |
| Yes | 189(17.2) | 412(16.0) |  |
| *Father’s characteristics* |  |  |  |
| BMI kg/m^2^ [Mean (SD)] | 23.2(3.5) | 23.5(3.6) | 0.056 |
| Education level [n (%)] |  |  | 0.008 |
| Junior high school or below | 178(16.2) | 189(12.8) |  |
| Senior middle school | 330(29.9) | 406(27.6) |  |
| Junior college | 295(26.8) | 400(27.2) |  |
| Bachelor degree or above | 299(27.1) | 477(32.4) |  |
| *Child characteristics* |  |  |  |
| Sex [n (%)] |  |  | 0.842 |
| Female | 548(49.7) | 725(49.3) |  |
| Male | 554(50.3) | 747(50.7) |  |
| Gestational age, wk [Mean (SD)] | 39.1(1.3) | 39.1(1.3) | 0.265 |
| Birth weight, g [Mean (SD)] | 3377.8(449.7) | 3369.8(419.0) | 0.639 |

**Table S2** Logistic regression analyses of maternal pregnancy-related anxiety and BMI trajectories in children from 48 to 72 months of age.

| **Time of maternal prenatal anxiety** | **Types of BMI Trajectories** | **Model 1** | | **Model 2** | | **Model 3** | |
| --- | --- | --- | --- | --- | --- | --- | --- |
|  |  | ***OR (95% CI)*** | ***P-value*** | ***OR (95% CI)*** | ***P-value*** | ***OR (95% CI)*** | ***P-value*** |
| First trimester |  |  |  |  |  |  |  |
|  | Normal BMI trajectory | Reference |  | Reference |  | Reference |  |
|  | High BMI trajectory | 0.90(0.69,1.17) | 0.439 | 0.88(0.67,1.15) | 0.880 | 0.88(0.68,1.16) | 0.369 |
|  | Very high trajectory | 1.18(0.75,1.86) | 0.482 | 1.17(0.74,1.86) | 0.507 | 1.14(0.72,1.82) | 0.580 |
| Second trimester |  |  |  |  |  |  |  |
|  | Normal BMI trajectory | Reference |  | Reference |  | Reference |  |
|  | High BMI trajectory | 0.93(0.75,1.16) | 0.538 | 0.93(0.75,1.16) | 0.527 | 0.93(0.75,1.17) | 0.539 |
|  | Very high trajectory | 0.95(0.64,1.40) | 0.797 | 0.95(0.64,1.41) | 0.788 | 0.93(0.63,1.39) | 0.729 |
| Third trimester |  |  |  |  |  |  |  |
|  | Normal BMI trajectory | Reference |  | Reference |  | Reference |  |
|  | High BMI trajectory | 0.82(0.66,1.03) | 0.083 | 0.82(0.65,1.02) | 0.079 | 0.82(0.65,1.02) | 0.079 |
|  | Very high trajectory | **0.54(0.34,0.84)** | 0.006 | **0.55(0.35,0.87)** | 0.010 | **0.53(0.34,0.84)** | 0.007 |
| At least one trimester during pregnancy |  |  |  |  |  |  |  |
|  | Normal BMI trajectory | Reference |  | Reference |  | Reference |  |
|  | High BMI trajectory | 0.88(0.71,1.08) | 0.205 | 0.86(0.70,1.06) | 0.159 | 0.87(0.70,1.07) | 0.185 |
|  | Very high trajectory | 1.01(0.70,1.46) | 0.960 | 1.00(0.69,1.46) | 0.998 | 0.98(0.67,1.43) | 0.914 |
| New-onset anxiety in the Second trimester |  |  |  |  |  |  |  |
|  | Normal BMI trajectory | Reference |  | Reference |  | Reference |  |
|  | High BMI trajectory | 0.93(0.65,1.33) | 0.674 | 0.93(0.65,1.35) | 0.716 | 0.93(0.64,1.35) | 0.709 |
|  | Very high trajectory | 0.53(0.25,1.13) | 0.102 | 0.54(0.25,1.17) | 0.116 | 0.50(0.23,1.09) | 0.080 |
| New-onset anxiety in the Third trimester |  |  |  |  |  |  |  |
|  | Normal BMI trajectory | Reference |  | Reference |  | Reference |  |
|  | High BMI trajectory | **0.63(0.41,0.97)** | 0.036 | **0.62(0.40,0.95)** | 0.029 | 0.65(0.42,1.01) | 0.053 |
|  | Very high trajectory | 0.55(0.23,1.29) | 0.170 | 0.55(0.23,1.32) | 0.181 | 0.50(0.21,1.22) | 0.127 |
| Both Second and Third trimesters |  |  |  |  |  |  |  |
|  | Normal BMI trajectory | reference |  | reference |  | reference |  |
|  | High BMI trajectory | 0.84(0.64,1.09) | 0.195 | 0.84(0.64,1.10) | 0.195 | 0.83(0.63,1.08) | 0.161 |
|  | Very high trajectory | **0.53(0.30,0.92)** | 0.023 | **0.54(0.31,0.93)** | 0.028 | **0.51(0.29,0.89)** | 0.019 |

Model 1: adjusted for maternal age, residence, race, family monthly income per capita, maternal education level, father’s education level, maternal pre-pregnancy BMI, father’s BMI, parity, maternal metabolic dysfunctions, alcohol use, and tobacco use during pregnancy.

Model 2: adjusted for covariates in model 1+ birth weight, gestational age, children’s sex

Model 3: adjusted for covariates in model 2+breastfeeding duration, main caregivers before 3 years, average outdoor activity time, parenting style score, and children’s diet.

**Table S3** Logistic regression analyses of maternal pregnancy-related anxiety and BF trajectories in children from 48 to 72 months of age.

| **Time of maternal prenatal anxiety** | **Types of BF trajectories** | **Model 1** | | **Model 2** | | **Model 3** | |
| --- | --- | --- | --- | --- | --- | --- | --- |
|  |  | ***OR (95% CI)*** | ***P-value*** | ***OR (95% CI)*** | ***P-value*** | ***OR (95% CI)*** | ***P-value*** |
| First trimester |  |  |  |  |  |  |  |
|  | Normal BF trajectory | Reference |  | Reference |  | Reference |  |
|  | High BF trajectory | 1.01(0.71,1.43) | 0.968 | 0.99(0.69,1.40) | 0.926 | 0.97(0.68,1.39) | 0.878 |
| Second trimester |  |  |  |  |  |  |  |
|  | Normal BF trajectory | Reference |  | Reference |  | Reference |  |
|  | High BF trajectory | 0.83(0.62,1.12) | 0.222 | 0.83(0.61,1.12) | 0.213 | 0.83(0.61,1.12) | 0.214 |
| Third trimester |  |  |  |  |  |  |  |
|  | Normal BF trajectory | Reference |  | Reference |  | Reference |  |
|  | High BF trajectory | **0.72(0.53,0.99)** | 0.043 | **0.73(0.53,1.00)** | 0.049 | **0.73(0.53,1.00)** | 0.049 |
| At least one trimester during pregnancy |  |  |  |  |  |  |  |
|  | Normal BF trajectory | Reference |  | Reference |  | Reference |  |
|  | High BF trajectory | 0.92(0.70,1.21) | 0.545 | 0.91(0.69,1.20) | 0.481 | 0.90(0.68,1.19) | 0.441 |
| New-onset anxiety in the Second trimester |  |  |  |  |  |  |  |
|  | Normal BF trajectory | Reference |  | Reference |  | Reference |  |
|  | High BF trajectory | 0.65(0.39,1.09) | 0.099 | 0.65(0.39,1.09) | 0.102 | 0.62(0.37,1.05) | 0.077 |
| New-onset anxiety in the Third trimester |  |  |  |  |  |  |  |
|  | Normal BF trajectory | Reference |  | Reference |  | Reference |  |
|  | High BF trajectory | 0.86(0.48,1.51) | 0.587 | 0.85(0.48,1.51) | 0.580 | 0.86(0.48,1.53) | 0.606 |
| Both Second and Third trimesters |  |  |  |  |  |  |  |
|  | Normal BF trajectory | Reference |  | Reference |  | Reference |  |
|  | High BF trajectory | **0.65(0.44,0.95)** | 0.026 | **0.65(0.44,0.96)** | 0.029 | **0.64(0.43,0.94)** | 0.024 |

Model 1: adjusted for maternal age, residence, race, family monthly income per capita, maternal education level, father’s education level, maternal pre-pregnancy BMI, father’s BMI, parity, maternal metabolic dysfunctions, alcohol use, and tobacco use during pregnancy.

Model 2: adjusted for covariates in model 1+ birth weight, gestational age, children’s sex

Model 3: adjusted for covariates in model 2+breastfeeding duration, main caregivers before 3 years, average outdoor activity time, parenting style score, and children’s diet.

**Table S4** Multiple linear regression analyses of maternal pregnancy-related anxiety and children’s BMI levels aged at 48 to 72 months.

| **Time of maternal prenatal anxiety** | Age of children | **Model 1** | ***P-value*** | **Model 2** | ***P-value*** | **Model 3** | ***P-value*** |
| --- | --- | --- | --- | --- | --- | --- | --- |
|  |  | ***β(95%CI)*** |  | ***β(95%CI)*** |  | ***β(95%CI)*** |  |
| First trimester |  |  |  |  |  |  |  |
|  | 48 months | -0.056(-0.256,0.145) | 0.587 | -0.076(-0.275,0.123) | 0.454 | -0.069(-0.270,0.131) | 0.496 |
|  | 54 months | 0.065(-0.134,0.263) | 0.521 | 0.037(-0.161,0.234) | 0.716 | 0.041(-0.158,0.239) | 0.687 |
|  | 60 months | 0.042(-0.165,0.249) | 0.691 | 0.024(-0.183,0.230) | 0.822 | 0.029(-0.179,0.236) | 0.787 |
|  | 66 months | 0.047(0.179,0.273) | 0.684 | 0.011(-0.213,0.235) | 0.925 | 0.010(-0.215,0.235) | 0.932 |
|  | 72 months | 0.100(-0.178,0.378) | 0.481 | 0.088(-0.184,0.360) | 0.527 | 0.089(-0.184,0.362) | 0.521 |
| Second trimester |  |  |  |  |  |  |  |
|  | 48 months | -0.121(-0.294,0.052) | 0.170 | -0.124(-0.295,0.047) | 0.155 | -0.124(-0.296,0.048) | 0.158 |
|  | 54 months | -0.034(-0.199,0.132) | 0.690 | -0.043(-0.207,0.121) | 0.605 | -0.045(-0.210,0.119) | 0.588 |
|  | 60 months | -0.011(-0.185,0.162) | 0.897 | -0.012(-0.184,0.160) | 0.890 | -0.015(-0.188,0.158) | 0.865 |
|  | 66 months | -0.076(-0.266,0.115) | 0.435 | -0.100(-0.288,0.088) | 0.296 | -0.098(-0.287,0.092) | 0.312 |
|  | 72 months | -0.072(-0.310,0.167) | 0.557 | -0.048(-0.282,0.186) | 0.689 | -0.040(-0.275,0.195) | 0.740 |
| Third trimester |  |  |  |  |  |  |  |
|  | 48 months | **-0.201(-0.373,-0.029)** | 0.022 | **-0.202(-0.373,-0.032)** | 0.020 | **-0.200(-0.372,-0.028)** | 0.023 |
|  | 54 months | -0.135(-0.303,0.033) | 0.115 | -0.131(-0.297,0.036) | 0.123 | -0.130(-0.298,0.037) | 0.126 |
|  | 60 months | **-0.217(-0.393,-0.042)** | 0.015 | **-0.206(-0.380,-0.032)** | 0.020 | **-0.213(-0.387,-0.038)** | 0.017 |
|  | 66 months | -0.173(-0.363,0.018) | 0.076 | -0.170(-0.358,0.018) | 0.076 | -0.168(-0.357,0.022) | 0.083 |
|  | 72 months | -0.099(-0.334,0.137) | 0.412 | -0.101(-0.332,0.131) | 0.393 | -0.083(-0.316,0.150) | 0.486 |
| At least one trimester during pregnancy |  |  |  |  |  |  |  |
|  | 48 months | -0.026(-0.185,0.132) | 0.745 | -0.034(-0.191,0.123) | 0.673 | -0.024(-0.183,0.135) | 0.766 |
|  | 54 months | 0.019(-0.134,0.172) | 0.807 | 0.003(-0.149,0.155) | 0.968 | 0.008(-0.146,0.161) | 0.920 |
|  | 60 months | -0.014(-0.177,0.149) | 0.866 | -0.021(-0.183,0.142) | 0.801 | -0.019(-0.183,0.145) | 0.823 |
|  | 66 months | -0.072(-0.249,0.105) | 0.424 | -0.105(-0.280,0.070) | 0.239 | -0.100(-0.277,0.076) | 0.266 |
|  | 72 months | 0.006(-0.213,0.224) | 0.960 | 0.001(-0.213,0.215) | 0.991 | 0.018(-0.198,0.234) | 0.868 |
| New-onset anxiety in the Second trimester |  |  |  |  |  |  |  |
|  | 48 months | -0.068(-0.345,0.210) | 0.632 | -0.057(-0.331,0.217) | 0.685 | -0.059(-0.335,0.217) | 0.676 |
|  | 54 months | -0.023(-0.276,0.230) | 0.857 | -0.013(-0.264,0.237) | 0.918 | -0.023(-0.275,0.230) | 0.860 |
|  | 60 months | -0.117(-0.397,0.162) | 0.410 | -0.102(-0.379,0.176) | 0.472 | -0.117(-0.396,0.162) | 0.412 |
|  | 66 months | -0.238(-0.543,0.067) | 0.125 | -0.214(-0.513,0.085) | 0.160 | -0.209(-0.510,0.092) | 0.173 |
|  | 72 months | -0.395(-0.787,0.003) | 0.056 | -0.317(-0.698,0.065) | 0.104 | -0.307(-0.691,0.077) | 0.117 |
| New-onset anxiety in the Third trimester |  |  |  |  |  |  |  |
|  | 48 months | -0.057(-0.381,0.266) | 0.728 | -0.075(-0.396,0.246) | 0.646 | -0.059(-0.383,0.265) | 0.722 |
|  | 54 months | -0.133(-0.434,0.167) | 0.384 | -0.139(-0.438,0.160) | 0.363 | -0.124(-0.427,0.178) | 0.420 |
|  | 60 months | -0.260(-0.585,0.066) | 0.118 | -0.253(-0.577,0.070) | 0.125 | -0.261(-0.589,0.066) | 0.117 |
|  | 66 months | -0.321(-0.689,0.046) | 0.087 | -0.344(-0.707,0.019) | 0.063 | -0.334(-0.700,0.032) | 0.074 |
|  | 72 months | -0.019(-0.446,0.408) | 0.932 | 0.093(-0.512,0.325) | 0.662 | -0.035(-0.457,0.388) | 0.872 |
| Both Second and Third trimesters |  |  |  |  |  |  |  |
|  | 48 months | **-0.244(-0.451,-0.038)** | 0.021 | **-0.247(-0.451,-0.042)** | 0.018 | **-0.256(-0.462,-0.050)** | 0.015 |
|  | 54 months | -0.122(-0.322,0.077) | 0.229 | -0.121(-0.319,0.076) | 0.227 | -0.131(-0.329,0.068) | 0.196 |
|  | 60 months | -0.193(-0.400,0.014) | 0.068 | -0.187(-0.391,0.019) | 0.076 | -0.195(-0.402,0.011) | 0.064 |
|  | 66 months | -0.178(-0.406,0.049) | 0.124 | -0.184(-0.407,0.039) | 0.106 | -0.185(-0.410,0.040) | 0.108 |
|  | 72 months | -0.174(-0.455,0.106) | 0.223 | -0.161(-0.435,0.114) | 0.252 | -0.154(-0.430,0.123) | 0.276 |

Model 1: adjusted for maternal age, residence, race, family monthly income per capita, maternal education level, father’s education level, maternal pre-pregnancy BMI, father’s BMI, parity, maternal metabolic dysfunctions, alcohol use, and tobacco use during pregnancy.

Model 2: adjusted for covariates in model 1+ birth weight, gestational age, children’s sex

Model 3: adjusted for covariates in model 2+breastfeeding duration, main caregivers before 3 years, average outdoor activity time, parenting style score, and children’s diet.

**Table S5** Multiple linear regression analyses of maternal pregnancy-related anxiety and children’s BF levels aged at 48 to 72 months.

| **Time of maternal prenatal anxiety** | Age of children | **Model 1** | ***P-value*** | **Model 2** | ***P-value*** | **Model 3** | ***P-value*** |
| --- | --- | --- | --- | --- | --- | --- | --- |
|  |  | ***β(95%CI)*** |  | ***β(95%CI)*** |  | ***β(95%CI)*** |  |
| First trimester |  |  |  |  |  |  |  |
|  | 48 months | -0.065(-0.260,0.130) | 0.514 | -0.082(-0.274, 0.110) | 0.403 | -0.070(-0.264,0.124) | 0.479 |
|  | 54 months | 0.066(-0.142,0.275) | 0.532 | 0.024(-0.186, 0.233) | 0.825 | 0.032(-0.176,0.240) | 0.763 |
|  | 60 months | 0.035(-0.244,0.314) | 0.804 | -0.086(-0.355, 0.183) | 0.529 | 0.027(-0253,0.308) | 0.849 |
|  | 66 months | 0.088(-0.179,0.356) | 0.517 | 0.037(-0.231, 0.305) | 0.785 | 0.045(-0.222,0.313) | 0.740 |
|  | 72 months | 0.051(-0.296,0.397) | 0.775 | 0.012(-0.335, 0.359) | 0.945 | 0.021(-0.325,0.367) | 0.904 |
| Second trimester |  |  |  |  |  |  |  |
|  | 48 months | -0.086(-0.246,0.074) | 0.290 | -0.068(-0.226, 0.090) | 0.398 | -0.093(-0.252,0.066) | 0.251 |
|  | 54 months | -0.133(-0.309,0.043) | 0.138 | -0.145(-0.321, 0.031) | 0.107 | -0.151(-0.326,0.023) | 0.089 |
|  | 60 months | -0.008(-0.232,0.217) | 0.947 | -0.076(-0.294, 0.143) | 0.498 | -0.015(-0.240,0.210) | 0.897 |
|  | 66 months | -0.106(-0.331,0.119) | 0.355 | -0.135(-0.360, 0.090) | 0.240 | -0.136(-0.361,0.088) | 0.233 |
|  | 72 months | -0.139(-0.437,0.159) | 0.360 | -0.117(-0.416, 0.182) | 0.444 | -0.118(-0.415,0.179) | 0.437 |
| Third trimester |  |  |  |  |  |  |  |
|  | 48 months | **-0.169(-0.330,-0.009)** | 0.038 | **-0.159(-0.318, -0.001)** | 0.049 | **-0.179(-0.339,-0.020)** | 0.028 |
|  | 54 months | **-0.232(-0.410,-0.054)** | 0.011 | **-0.232(-0.411, -0.054)** | 0.011 | **-0.251(-0.428,-0.074)** | 0.006 |
|  | 60 months | **-0.278(-0.506,-0.050)** | 0.017 | **-0.354(-0.575, -0.132)** | 0.002 | **-0.291(-0.520,-0.062)** | 0.013 |
|  | 66 months | -0.198(-0.422,0.026) | 0.084 | -0.198(-0.432, 0.028) | 0.086 | -0.207(-0.431,0.017) | 0.070 |
|  | 72 months | -0.087(-0.382,0.208) | 0.562 | -0.096(-0.393, 0.201) | 0.524 | -0.084(-0.379,0.211) | 0.579 |
| At least one trimester during pregnancy |  |  |  |  |  |  |  |
|  | 48 months | -0.022(-0.174,0.130) | 0.286 | -0.035(-0.185,0.116) | 0.653 | -0.024(-0.176,0.128) | 0.761 |
|  | 54 months | -0.124(-0.285,0.038) | 0.133 | -0.135(-0.295,0.026) | 0.100 | -0.152(-0.314,0.010) | 0.066 |
|  | 60 months | -0.026(-0.246,0.193) | 0.815 | -0.026(-0.246,0.194) | 0.817 | -0.039(-0.260,0.183) | 0.731 |
|  | 66 months | -0.064(-0.274,0.146) | 0.550 | -0.096(-0.305,0.112) | 0.365 | -0.103(-0.314,0.107) | 0.336 |
|  | 72 months | 0.057(-0.216,0.329) | 0.683 | 0.045(-0.226,0.316) | 0.745 | 0.061(-0.213,0.335) | 0.662 |
| New-onset anxiety in the Second trimester |  |  |  |  |  |  |  |
|  | 48 months | -0.014(-0.292,0.265) | 0.923 | -0.009(-0.282, 0.265) | 0.951 | -0.028(-0.305,0.249) | 0.843 |
|  | 54 months | **-0.291(-0.558,-0.025)** | 0.032 | **-0.276(-0.542, -0.009)** | 0.042 | **-0.328(-0.592,-0.063)** | 0.015 |
|  | 60 months | -0.148(-0.525,0.228) | 0.439 | -0.157(-0.535, 0.222) | 0.417 | -0.160(-0.537,0.218) | 0.406 |
|  | 66 months | -0.241(-0.601,0.118) | 0.188 | -0.225(-0.583, 0.133) | 0.218 | -0.246(-0.604,0.111) | 0.176 |
|  | 72 months | -0.331(-0.819,0.157) | 0.183 | -0.282(-0.772, 0.208) | 0.259 | -0.266(-0.752,0.221) | 0.284 |
| New-onset anxiety in the Third trimester |  |  |  |  |  |  |  |
|  | 48 months | -0.051(-0.371,0.270) | 0.757 | -0.055(-0.373, 0.262) | 0.732 | -0.054(-0.375,0.266) | 0.332 |
|  | 54 months | -0.315(-0.637,0.006) | 0.055 | -0.317(-0.642, 0.009) | 0.057 | **-0.357(-0.680,-0.034)** | 0.030 |
|  | 60 months | -0.352(-0.797,0.093) | 0.120 | -0.365(-0.815, 0.086) | 0.112 | -0.382(-0.832,0.067) | 0.095 |
|  | 66 months | -0.344(-0.775,0.086) | 0.117 | -0.348(-0.786, 0.089) | 0.118 | -0.381(-0.812,0.050) | 0.083 |
|  | 72 months | 0.195(-0.336,0.726) | 0.472 | 0.141(-0.400, 0.681) | 0.610 | 0.184(-0.350,0.717) | 0.499 |
| Both Second and Third trimesters |  |  |  |  |  |  |  |
|  | 48 months | **-0.203(-0.397,-0.009)** | 0.041 | **-0.194(-0.385, -0.002)** | 0.048 | **-0.221(-0.414,-0.028)** | 0.025 |
|  | 54 months | **-0.231(-0.444,-0.018)** | 0.033 | **-0.237(-0.450, -0.024)** | 0.029 | **-0.258(-0.469,-0.047)** | 0.017 |
|  | 60 months | -0.228(-0.503,0.048) | 0.105 | **-0.345(-0.611, -0.080)** | 0.011 | -0.238(-0.514,0.038) | 0.092 |
|  | 66 months | -0.200(-0.467,0.066) | 0.140 | -0.215(-0.481, 0.052) | 0.114 | -0.222(-0.487,0.043) | 0.101 |
|  | 72 months | -0.249(-0.598,0.101) | 0.163 | -0.243(-0.594, 0.107) | 0.174 | -0.241(-0.589,0.108) | 0.176 |

Model 1: adjusted for maternal age, residence, race, family monthly income per capita, maternal education level, father’s education level, maternal pre-pregnancy BMI, father’s BMI, parity, maternal metabolic dysfunctions, alcohol use, and tobacco use during pregnancy.

Model 2: adjusted for covariates in model 1+ birth weight, gestational age, children’s sex

Model 3: adjusted for covariates in model 2+breastfeeding duration, main caregivers before 3 years, average outdoor activity time, parenting style score, and children’s diet.

**Table S6** Logistic regression analyses of maternal pregnancy-related anxiety and weight status in children from 48 to 72 months of age.

| **Time of maternal prenatal anxiety** | Age of children | **Adjusted Model^✝^** | | | |
| --- | --- | --- | --- | --- | --- |
|  |  | **Underweight** | **Overweight** | **Obesity** | **Overweight&**  **Obesity** |
|  |  | ***OR (95% CI)*** | ***OR (95% CI)*** | ***OR (95% CI)*** | ***OR (95% CI)*** |
| First trimester |  |  |  |  |  |
|  | 48 months | 1.13(0.76,1.67) | 0.96(0.69,1.33) | 0.72(0.43,1.18) | 0.87(0.65,1.16) |
|  | 54 months | 0.93(0.61,1.40) | 0.90(0.60,1.36) | 1.19(0.67,2.12) | 0.99(0.70,1.40) |
|  | 60 months | 0.97(0.67,1.39) | 1.04(0.71,1.54) | 0.89(0.49,1.62) | 1.00(0.71,1.40) |
|  | 66 months | 0.97(0.66,1.42) | 1.25(0.85,1.83) | 1.11(0.64,1.92) | 1.21(0.86,1.68) |
|  | 72 months | 0.78(0.48,1.27) | 1.21(0.83,1.77) | 0.90(0.55,1.45) | 1.12(0.81,1.55) |
| Second trimester |  |  |  |  |  |
|  | 48 months | 0.96(0.67,1.36) | 0.81(0.61,1.07) | 0.74(0.50,1.11) | **0.79(0.62,1.00)^*^** |
|  | 54 months | 0.78(0.56,1.11) | 0.89(0.65,1.22) | 0.86(0.54,1.38) | 0.91(0.69,1.20) |
|  | 60 months | 0.98(0.72,1.33) | 0.97(0.70,1.34) | 0.95(0.59,1.52) | 0.96(0.73,1.28) |
|  | 66 months | 0.96(0.71,1.30) | 0.89(0.63,1.24) | 0.84(0.53,1.33) | 0.88(0.66,1.16) |
|  | 72 months | 0.79(0.53,1.18) | 1.03(0.74,1.44) | 0.83(0.55,1.25) | 0.98(0.74,1.30) |
| Third trimester |  |  |  |  |  |
|  | 48 months | 1,02(0.72,1.44) | **0.75(0.56,0.99)^*^** | 0.70(0.47,1.06)^#^ | **0.73(0.57,0.94)^*^** |
|  | 54 months | 0.72(0.51,1.03)^#^ | 0.74(0.53,1.03)^#^ | **0.59(0.35,1.00)^*^** | **0.73(0.54,0.97)^*^** |
|  | 60 months | 0.76(0.55,1.06) | **0.66(0.46,0.94)^*^** | **0.48(0.27,0.84)^**^** | **0.63(0.46,0.86)^**^** |
|  | 66 months | 0.91(0.67,1.24) | 0.85(0.60,1.19) | **0.60(0.36,0.99)^*^** | 0.77(0.58,1.04)^#^ |
|  | 72 months | 0.81(0.55,1.20) | 1.03(0.73,1.43) | 0.79(0.52,1.22) | 0.96(0.72,1.28) |
| At least one trimester during pregnancy |  |  |  |  |  |
|  | 48 months | 0.96(0.70,1.34) | 0.89(0.69,1.15) | 0.88(0.61,1.27) | 0.89(0.72,1.11) |
|  | 54 months | 0.80(0.58,1.11) | 0.90(0.66,1.22) | 1.11(0.71,1.74) | 0.99(0.76,1.28) |
|  | 60 months | 0.83(0.62,1.12) | 0.82(0.61,1.12) | 0.88(0.56,1.38) | 0.87(0.66,1.13) |
|  | 66 months | 1.02(0.76,1.36) | 0.99(0.73,1.34) | 0.84(0.55,1.30) | 0.94(0.72,1.22) |
|  | 72 months | 0.85(0.59,1.23) | 1.01(0.74,1.38) | 0.96(0.66,1.39) | 1.01(0.78,1.31) |
| New-onset anxiety in the Second trimester |  |  |  |  |  |
|  | 48 months | 0.82(0.52,1.29) | 0.71(0.36,1.39) | 0.67(0.35,1.27) | 0.81(0.54,1.20) |
|  | 54 months | 0.68(0.37,1.25) | 0.89(0.53,1.49) | 0.76(0.34,1.72) | 0.89(0.56,1.40) |
|  | 60 months | 0.74(0.44,1.26) | 0.63(0.36,1.12) | 0.55(0.24,1.30) | 0.63(0.38,1.04)^#^ |
|  | 66 months | 1.00(0.60,1.65) | 0.65(0.36,1.16) | 0.65(0.30,1.41) | 0.65(0.40,1.06)^#^ |
|  | 72 months | 0.68(0.33,1.38) | 0.73(0.41,1.30) | 0.47(0.22,1.02)^#^ | 0.65(0.40,1.07)^#^ |
| New-onset anxiety in the Third trimester |  |  |  |  |  |
|  | 48 months | 0.58(0.50,1.47) | 1.26(0.65,2.46) | 1.36(0.75,2.49) | 0.94(0.61,1.47) |
|  | 54 months | 0.92(0.49,1.75) | 0.73(0.37,1.43) | 1.16(0.48,2.83) | 0.86(0.49,1.50) |
|  | 60 months | 0.74(0.40,1.37) | **0.47(0.23,0.97)^*^** | 0.69(0.27,1.74) | 0.56(0.31,1.03)^#^ |
|  | 66 months | 1.22(0.69,2.16) | 0.90(0.48,1.71) | 0.38(0.11,1.31) | 0.71(0.40,1.28) |
|  | 72 months | 1.11(0.56,2.21) | 0.81(0.42,1.57) | 1.23(0.62,2.45) | 0.96(0.57,1.61) |
| Both Second and Third trimesters |  |  |  |  |  |
|  | 48 months | 1.00(0.65,1.52) | 0.71(0.51,1.01)^#^ | **0.57(0.34,0.97)^*^** | **0.67(0.50,0.90)^**^** |
|  | 54 months | 0.69(0.45,1.05) | 0.73(0.49,1.08) | 0.56(0.30,1.07) | **0.70(0.50,1.00)^*^** |
|  | 60 months | 0.86(0.58,1.25) | 0.72(0.48,1.09) | **0.43(0.21,0.90)^*^** | **0.65(0.45,0.94)^*^** |
|  | 66 months | 0.95(0.66,1.37) | 0.78(0.52,1.18) | 0.66(0.37,1.18) | 0.75(0.53,1.06) |
|  | 72 months | 0.71(0.43,1.16) | 1.02(0.69,1.51) | 0.61(0.34,1.07) | 0.90(0.64,1.28) |

**P<0.01; *P<0.05; ^#^P<0.10.

**^✝^**normal weight as a reference.

Adjusted Model: adjusted for maternal age, residence, race, family monthly income per capita, maternal education level, father’s education level, maternal pre-pregnancy BMI, father’s BMI, parity, maternal metabolic dysfunctions, alcohol use, and tobacco use during pregnancy.

**Table S7** Logistic regression analyses of maternal pregnancy-related anxiety and BMI z-score trajectories in children from 48 to 72 months of age.

| **Time of maternal prenatal anxiety** | **Types of BMI z-score Trajectories** | **Model 1** | | **Model 2** | | **Model 3** | |
| --- | --- | --- | --- | --- | --- | --- | --- |
|  |  | ***OR (95% CI)*** | ***P-value*** | ***OR (95% CI)*** | ***P-value*** | ***OR (95% CI)*** | ***P-value*** |
| First trimester |  |  |  |  |  |  |  |
|  | Low BMI trajectory | 0.91(0.69,1.19) | 0.486 | 0.93(0.71,1.22) | 0.598 | 0.91(0.69,1.20) | 0.495 |
|  | Normal BMI trajectory | Reference |  | Reference |  | Reference |  |
|  | High BMI trajectory | 1.04(0.72,1.51) | 0.848 | 1.04(0.71,1.51) | 0.847 | 1.03(0.70,1.50) | 0.900 |
| Second trimester |  |  |  |  |  |  |  |
|  | Low BMI trajectory | 0.86(0.69,1.08) | 0.187 | 0.86(0.68,1.07) | 0.179 | 0.84(0.67,1.06) | 0.142 |
|  | Normal BMI trajectory | Reference |  | Reference |  | Reference |  |
|  | High BMI trajectory | 0.81(0.59,1.12) | 0.201 | 0.81(0.59,1.12) | 0.198 | 0.80(0.58,1.11) | 0.184 |
| Third trimester |  |  |  |  |  |  |  |
|  | Low BMI trajectory | 0.94(0.75,1.18) | 0.614 | 0.95(0.76,1.19) | 0.664 | 0.93(0.74,1.16) | 0.511 |
|  | Normal BMI trajectory | Reference |  | Reference |  | Reference |  |
|  | High BMI trajectory | **0.69(0.49,0.97)** | 0.032 | **0.71(0.50,1.00)** | 0.050 | **0.70(0.50,0.99)** | 0.044 |
| At least one trimester during pregnancy |  |  |  |  |  |  |  |
|  | Low BMI trajectory | 0.95(0.77,1.17) | 0.629 | 0.95(0.77,1.18) | 0.664 | 0.93(0.75,1.15) | 0.497 |
|  | Normal BMI trajectory | Reference |  | Reference |  | Reference |  |
|  | High BMI trajectory | 0.96(0.72,1.29) | 0.804 | 0.97(0.72,1.30) | 0.814 | 0.96(0.71,1.30) | 0.791 |
| New-onset anxiety in the Second trimester |  |  |  |  |  |  |  |
|  | Low BMI trajectory | 0.86(0.59,1.25) | 0.429 | 0.84(0.58,1.24) | 0.381 | 0.82(0.56,1.20) | 0.303 |
|  | Normal BMI trajectory | Reference |  | Reference |  | Reference |  |
|  | High BMI trajectory | **0.57(0.32,0.99)** | 0.047 | 0.57(0.33,1.01) | 0.055 | **0.55(0.31,0.97)** | 0.040 |
| New-onset anxiety in the Third trimester |  |  |  |  |  |  |  |
|  | Low BMI trajectory | **1.56(1.02,2.39)** | 0.038 | **1.60(1.04,2.44)** | 0.032 | 1.48(0.96,2.27) | 0.078 |
|  | Normal BMI trajectory | Reference |  | Reference |  | Reference |  |
|  | High BMI trajectory | 1.19(0.66,2.16) | 0.566 | 1.20(0.66,2.18) | 0.557 | 1.18(0.64,2.17) | 0.592 |
| Both Second and Third trimesters |  |  |  |  |  |  |  |
|  | Low BMI trajectory | 0.85(0.65,1.11) | 0.224 | 0.85(0.65,1.12) | 0.240 | 0.83(0.63,1.09) | 0.187 |
|  | Normal BMI trajectory | Reference |  | Reference |  | Reference |  |
|  | High BMI trajectory | **0.58(0.38,0.89)** | 0.012 | **0.60(0.39,0.91)** | 0.017 | **0.57(0.38,0.88)** | 0.011 |

Model 1: adjusted for maternal age, residence, race, family monthly income per capita, maternal education level, father’s education level, maternal pre-pregnancy BMI, father’s BMI, parity, maternal metabolic dysfunctions, alcohol use, and tobacco use during pregnancy.

Model 2: adjusted for covariates in model 1+ birth weight, gestational age, children’s sex

Model 3: adjusted for covariates in model 2+breastfeeding duration, main caregivers before 3 years, average outdoor activity time, parenting style score, and children’s diet.

**Table S8** Logistic regression analyses of maternal pregnancy-related anxiety and BF z-score trajectories in children from 48 to 72 months of age.

| **Time of maternal prenatal anxiety** | **Types of BF z-score trajectories** | **Model 1** | | **Model 2** | | **Model 3** | |
| --- | --- | --- | --- | --- | --- | --- | --- |
|  |  | ***OR (95% CI)*** | ***P-value*** | ***OR (95% CI)*** | ***P-value*** | ***OR (95% CI)*** | ***P-value*** |
| First trimester |  |  |  |  |  |  |  |
|  | Normal BF trajectory | Reference |  | Reference |  | Reference |  |
|  | High BF trajectory | 1.00(0.70,1.43) | 0.993 | 0.97(0.68,1.39) | 0.881 | 0.96(0.67,1.37) | 0.821 |
| Second trimester |  |  |  |  |  |  |  |
|  | Normal BF trajectory | Reference |  | Reference |  | Reference |  |
|  | High BF trajectory | 0.84(0.62,1.13) | 0.247 | 0.83(0.62,1.13) | 0.239 | 0.83(0.61,1.12) | 0.225 |
| Third trimester |  |  |  |  |  |  |  |
|  | Normal BF trajectory | Reference |  | Reference |  | Reference |  |
|  | High BF trajectory | **0.65(0.47,0.89)** | 0.008 | **0.65(0.47,0.90)** | 0.009 | **0.64(0.46,0.89)** | 0.008 |
| At least one trimester during pregnancy |  |  |  |  |  |  |  |
|  | Normal BF trajectory | Reference |  | Reference |  | Reference |  |
|  | High BF trajectory | 0.91(0.67,1.20) | 0.500 | 0.89(0.67,1.18) | 0.423 | 0.88(0.67,1.17) | 0.391 |
| New-onset anxiety in the Second trimester |  |  |  |  |  |  |  |
|  | Normal BF trajectory | Reference |  | Reference |  | Reference |  |
|  | High BF trajectory | 0.67(0.40,1.12) | 0.126 | 0.67(0.40,1.12) | 0.125 | 0.63(0.37,1.07) | 0.084 |
| New-onset anxiety in the Third trimester |  |  |  |  |  |  |  |
|  | Normal BF trajectory | Reference |  | Reference |  | Reference |  |
|  | High BF trajectory | 0.76(0.42,1.37) | 0.359 | 0.74(0.41,1.35) | 0.322 | 0.75(0.41,1.37) | 0.341 |
| Both Second and third trimesters |  |  |  |  |  |  |  |
|  | Normal BF trajectory | Reference |  | Reference |  | Reference |  |
|  | High BF trajectory | **0.61(0.41,0.90)** | 0.012 | **0.61(0.41,0.91)** | 0.014 | **0.59(0.40,0.88)** | 0.009 |

Model 1: adjusted for maternal age, residence, race, family monthly income per capita, maternal education level, father’s education level, maternal pre-pregnancy BMI, father’s BMI, parity, maternal metabolic dysfunctions, alcohol use, and tobacco use during pregnancy.

Model 2: adjusted for covariates in model 1+ birth weight, gestational age, children’s sex

Model 3: adjusted for covariates in model 2+breastfeeding duration, main caregivers before 3 years, average outdoor activity time, parenting style score, and children’s diet.


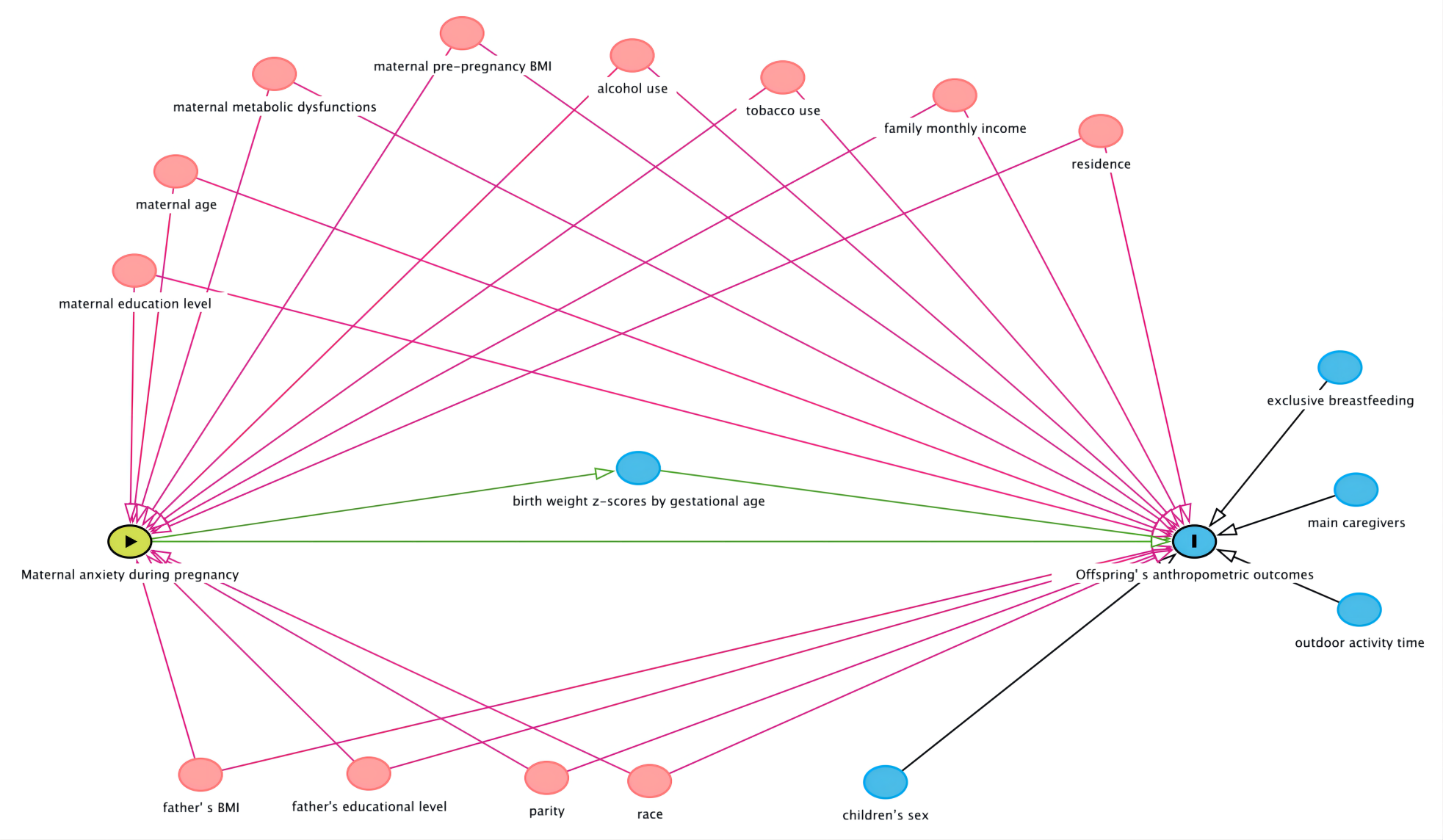


**Figure S1** Directed acyclic graph of the relationship between maternal pregnancy-related anxiety and early childhood weight status.


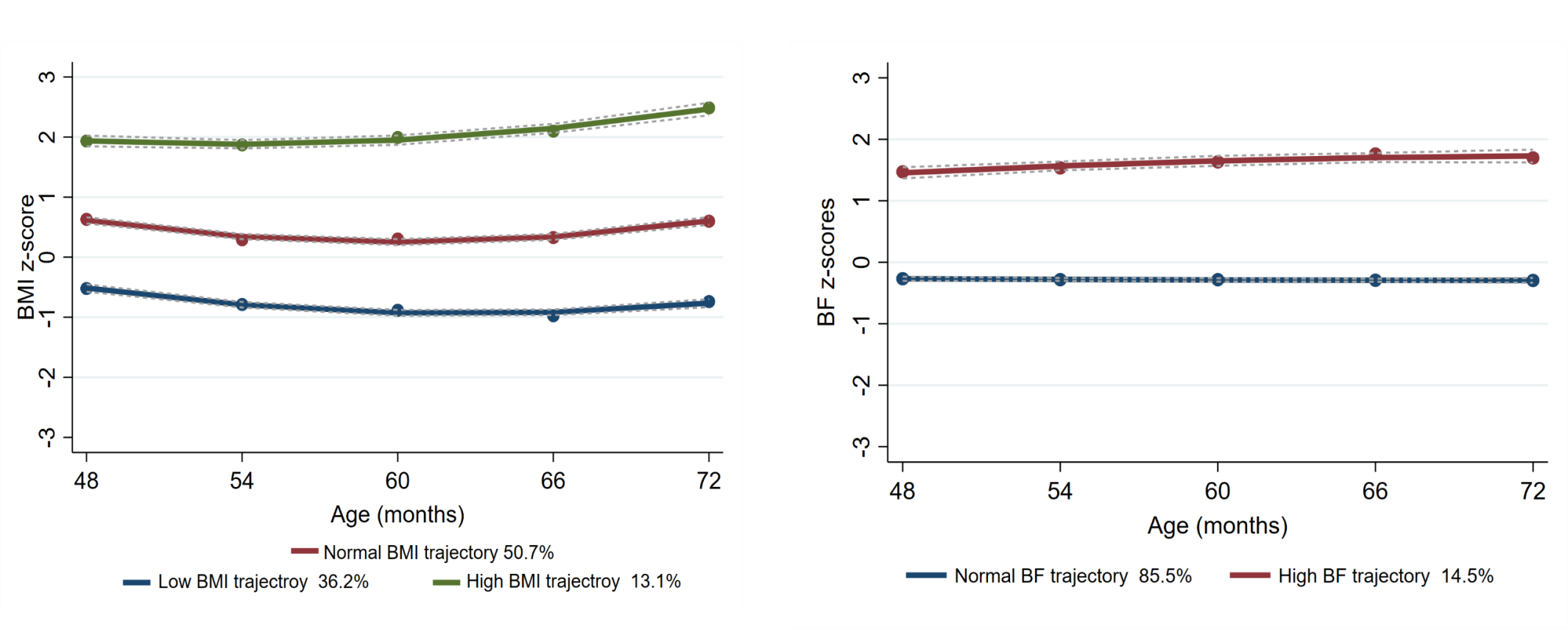


**Figure S2** BMI z-score trajectories and BF z-scores of children from 48 to 72 months.
